# Incidence of central precocious puberty more than doubled during COVID-19 pandemic: Single-center retrospective review in the United States

**DOI:** 10.3389/fped.2022.1007730

**Published:** 2022-11-30

**Authors:** Marcela Vargas Trujillo, Tiranun Rungvivatjarus, Karen O. Klein

**Affiliations:** ^1^ Department of Pediatrics, University of California, San Diego, San Diego, CA, United States; ^2^ Department of Pediatrics, Rady Children's Hospital-San Diego, University of California, San Diego, San Diego, CA, United States

**Keywords:** central precocious puberty, COVID-19, incidence, early puberty, coronavirus

## Abstract

**Background and aim of the study:**

The frequency of new visits for precocious puberty increased during the Covid-19 pandemic in the pediatric endocrinology clinic at Rady Children's Hospital in San Diego, CA, US. A few recent studies have shown an increase in the frequency of Central Precocious Puberty (CPP) in other centers during this pandemic. This study evaluated the change in incidence of new CPP cases requiring treatment with GnRH agonist (GnRHa) at Rady Children's Hospital during the Covid-19 pandemic and compared it to pre-pandemic years.

**Methods:**

Data were reviewed retrospectively to compare the number of visits of children newly diagnosed with CPP treated with GnRHa during the Covid-19 pandemic (5/2020–4/2021) and before the pandemic (5/2018–4/2019). Clinical and bone maturation data were evaluated as well as differences in timing from diagnosis to onset of treatment. The incidence of CPP requiring treatment for 5 years prior to the pandemic was also reviewed to evaluate for trends over time.

**Results:**

A total of 92 subjects were included. During pre-Covid year, 28 children (1 boy, 27 girls) were treated with GnRHa for CPP out of 2,340 new endocrinology visits (1.2% of patients seen). During Covid-19 year, 64 children (7 boys, 57 girls) were treated out of 2,261 new visits (2.8%). The incidence of new CPP cases requiring GnRHa during the pandemic more than doubled compared to pre-pandemic. Age at onset of treatment, degree of bone age (BA) advancement, time from diagnosis to onset of treatment, and changes in BMI during the pandemic were not different from pre-pandemic.

**Conclusion:**

CPP cases requiring GnRHa treatment significantly increased during the first year of the Covid-19 pandemic. This was not related to increased BMI or delay in onset of treatment. Age at diagnosis, degree of bone age advancement, and time from diagnosis to onset of treatment were all similar during the first year of the pandemic compared to the prior year.

## Introduction

CPP occurs when the hypothalamic-pituitary-gonadal axis is activated prematurely, resulting in increased gonadal steroid hormone production. Children with this condition develop secondary sexual characteristics prematurely (before the age of 8 and 9 years in girls and boys, respectively) and can experience accelerated bone maturation and premature epiphyseal fusion, which results in reduced adult stature. Children with CPP can benefit from gonadotropin releasing hormone agonist (GnRHa) treatment to stop the progression of puberty and accelerated growth, and consequently improve final height (1).

The subjective impression was that there were more new patients evaluated for precocious puberty at Rady Children's Hospital since the Covid-19 pandemic started. Moreover, it seemed like an increased number of those who presented for initial evaluation met criteria for the diagnosis of CPP and required treatment with GnRHa. On review of the literature, it became apparent that other centers were having an increase in incidence of CPP cases during the pandemic (2–8). This study was conducted to assess if the subjective impression of higher incidence of CPP cases during the pandemic was substantiated and significant. In addition, the present study evaluated differences in age at diagnosis, degree of bone maturation, BMI, and differences in the timing from diagnosis to treatment initiation during the pandemic compared to the year prior.

## Methods

### Study design and setting

This is a single-center retrospective comparison of the incidence of newly diagnosed children with CPP requiring GnRHa treatment during the Covid-19 pandemic (5/2020–4/2021) and pre-Covid-19 year (5/2018–4/2019). Incidence data for the second year of the pandemic, between 5/2021 and 2/2022 were also analyzed. In addition, the incidence of CPP during the previous 3 years pre-pandemic (5/2015–4/2018) were analyzed, to look for overall trends in incidence unrelated to Covid-19. The study was conducted at a free-standing, tertiary care academic children's hospital and pediatric endocrinology specialty outpatient clinic in San Diego, California. Patients with suspected CPP are referred to endocrinology clinic by general pediatricians and other subspecialists practicing in the San Diego County and surrounding areas. Subjects were identified by electronic medical record (EMR) query (Epic Systems Corporation, Verona, Wisconsin). A new diagnosis of CPP was defined as having all of the following: (1) at least 1 endocrinology clinic visit associated with one of the following 4 CPP ICD codes: early puberty, premature thelarche, precocious puberty, or central precocious puberty; (2) chronological age <8 years for girls and <9 years for boys at onset of symptoms; (3) a random luteinizing hormone (LH) level >0.3 IU/L (9), or a GnRH-stimulated peak LH level >5 IU/L (10), or a GnRH-stimulated estradiol >40 pg/ml (11, 12). GnRHa stimulation tests were performed by administering aqueous leuprolide acetate subcutaneously, at a standard dose of 20 mcg/kg (maximum dose 500 mcg). Blood samples were obtained at 1 h for measurement of LH level and at 18–24 h for measurement of estradiol in girls or testosterone in boys. In addition, only patients who received GnRHa treatment were included Date of diagnosis was defined by date of confirmatory blood test. Patient characteristics and outcome variables were obtained by EMR query and manual chart review (KK, MVT). The University of California San Diego (UCSD) Institutional Review Board approved this study, and a waiver of informed consent was granted for the collection of retrospective data.

The primary outcome measure was the proportion of patients newly diagnosed with CPP during the Covid-19 pandemic compared to the pre-pandemic period. Newly diagnosed CPP patients were defined as those whose visit was associated with new CPP diagnosis and later received GnRHa treatment. Bone age (BA), BMI, time from diagnosis to GnRHa order, and time from GnRHa order to first day of treatment were evaluated. GnRHa included leuprolide, triptorelin, and histrelin. First day of GnRHa treatment was captured by the date of the first endocrinology nurse visit for GnRHa administration. During this time period, there was only one patient who received histrelin, and first day of treatment was captured as day of insertion.

### Analysis

Descriptive analysis used means ± standard deviation (SD). Chi-square was used for comparing proportions of newly diagnosed patients with CPP during pre-Covid-19 years and during Covid-19 pandemic year. Two-tailed Student T-tests were used to compare variables between groups. Data regarding Covid-19 cases per month in California and in San Diego County were obtained from Government data (13) for children under 18 years of age. San Diego infection rates paralleled those of California, so California data was used for more robust statistics and divided by 3,000 for visual scaling.

## Results

### Incidence of patients diagnosed with CPP and treated with GnRHa

There were 2,340 new patients seen during pre-Covid-19 years, including all chief complaints. Of these, 28 children (1 boy, 27 girls) were diagnosed with CPP and treated with GnRHa (1.2% of patients seen). During Covid-19 year, 64 children (7 boys, 57 girls) were diagnosed with CPP and treated with GnRHa, out of 2,261 new visits (2.8% of patients seen). The incidence of new CPP cases requiring GnRHa more than doubled during Covid compared to the pre-Covid years (*p* < 0.01, Chi Square).

The percentage of boys was higher in Covid-19 year at 7 cases, compared to an average of 2.5 boys during pre-covid years. The range was broad, however, ranging from 1 to 5 boys per year over those years.

**Figure 1:**
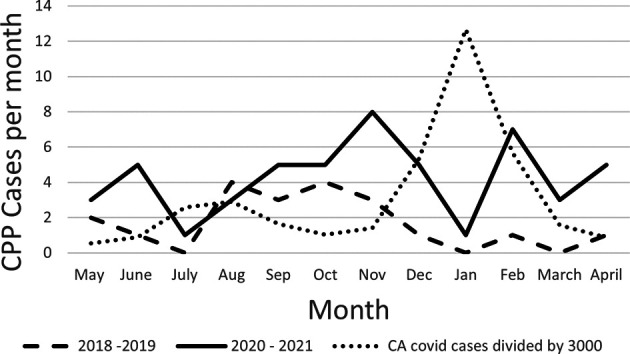
CPP cases by month of diagnosis. Dashed line represents pre-pandemic, solid line represents Covid-19, dotted line represents Covid cases in California divided by 3,000 for visual scaling.

The incidence of CPP over the 5 years prior to Covid-19 averaged 30 ± 5 cases/year with no trend over time, which is similar to 28 cases/year seen in the pre-pandemic year included in our study (5/2018–4/2019). The incidence of patients with CPP requiring GnRHa treatment continues to be tracked and 58 new cases have been identified between 5/2021–2/2022 (second year of Covid-19), which would be equivalent to 69 cases annually, similar to the first pandemic year analyzed (Figure 1).

### Patient demographics

The age at diagnosis was 7.1 ± 2.1 (1.8–10.0) years in pre-Covid-19 years, and 7.6 ± 1.43 (1.5–10.5) years during Covid-19 year (*p* = 0.54). BMI in pre-Covid-19 years was 17.6 ± 2.8 (12.7–25.5) and 18.2 ± 3.5 (11.4–29.4) during Covid-19 (*p* = 0.69). BA at diagnosis was 9.5 ± 2.6 (1.6–13.0) in the pre-Covid-19 group and 9.5 ± 1.7 (2.5–12.6) in the Covid-19 group (*p* = 0.66). Likewise, BA/CA (chronological age) and BA-CA did not show a statistical significance between the two groups (*p* = 0.38 and 0.28, respectively) (Table 1).

**Table 1:** Demographics.

	Age Dx (years)	BMI (kg/m^2^)	BA (years)	BA/CA	BA-CA (years)
Pre-Covid-19	7.1 ± 2.1 (1.8–10.0)	17.6 ± 2.8 (12.7–25.5)	9.5 ± 2.6 (1.6–13.0)	1.3 ± 0.2 (1.0–2.0)	2.3 ± 1.1 (0–5.0)
Covid-19	7.60 ± 1.43 (1.5–10.5)	18.2 ± 3.5 (11.4–29.4)	9.5 ± 1.7 (2.5–12.6)	1.3 ± 0.3 (0.9–3.3)	1.9 ± 1.3 (−0.7 to 7.0)
*p*-value	0.54	0.69	0.66	0.38	0.28

Mean ± SD (range), Dx, diagnosis; BMI, body mass index; BA, bone age; CA, chronological age.

### Days from diagnosis to treatment order and to start of treatment

In the pre-Covid-19 years there was a time range of 94.2 ± 109.2 (4.0–397.0) days from diagnosis to the order of GnRHa and 54 ± 62 days from the order to the first injection; and during Covid-19, the time range was 65.70 ± 77.8 (0–306) days between diagnosis and order, and 62 ± 65 days between order and treatment onset. The time range was broad for both groups and the means are not significantly different (Table 2).

**Table 2:** Age at diagnosis and time to treatment order and 1st injection.

	Days from Dx to order	Days from order to 1st treatment
Pre-Covid-19	94.2 ± 109.2 (4.0–397.0)	54 ± 62 (4.0–287.0)
Covid-19	65.70 ± 77.8 (0–306.0)	62 ± 65 (5.0–285.0)

Mean ± SD (range), Dx, diagnosis.

### CPP incidence and COVID-19 peaks in the community

**Figure 2:**
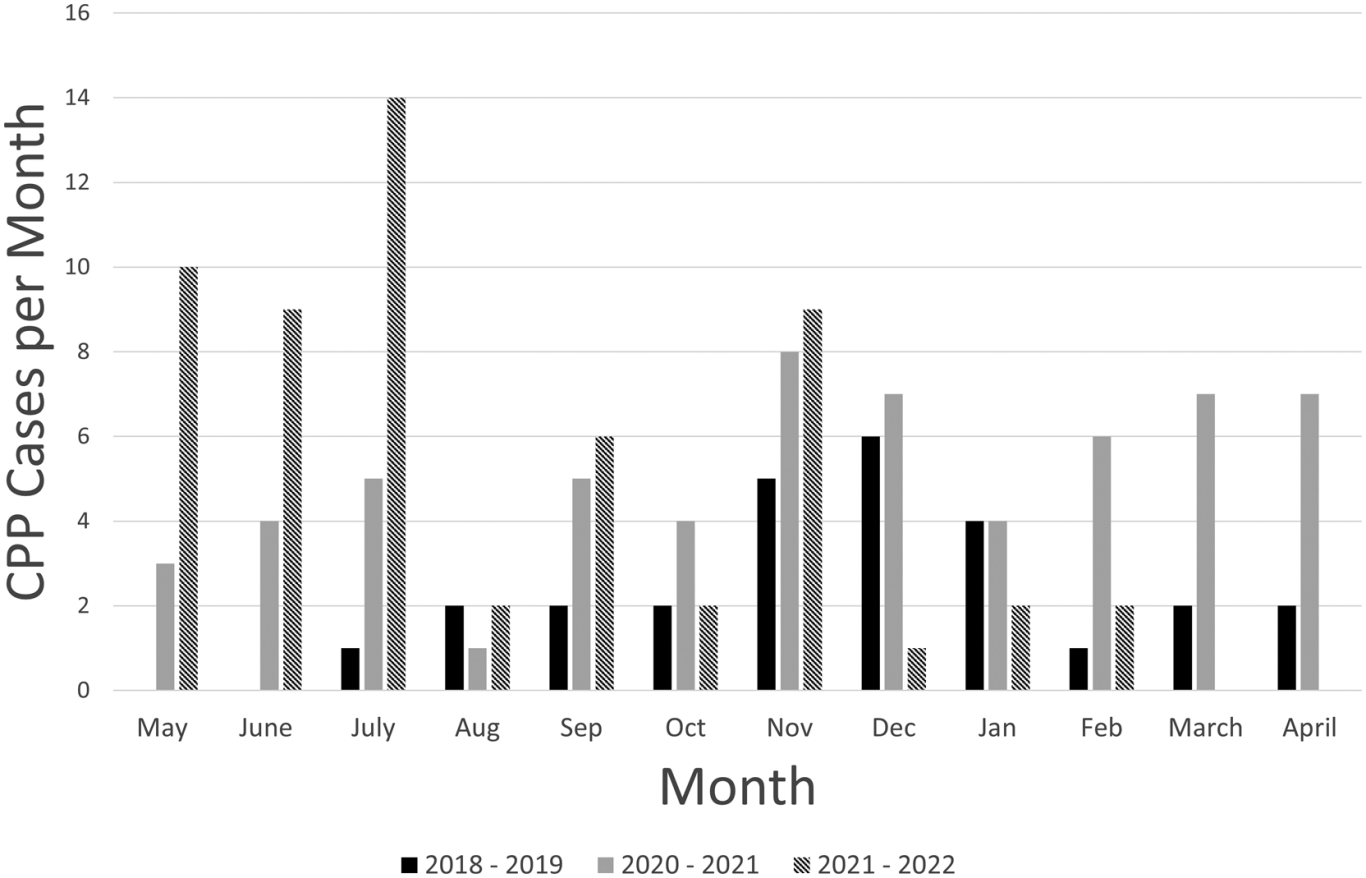
CPP cases by month of diagnosis, per year (from May to April of each year shown). Solid black bars represent pre-pandemic year, grey bars represent Covid-19 year, hatched bars represent the year after Covid-19.

The number of cases per month appeared to increase approximately 5–6 months after the highest peak of Covid-19 cases (Figure 2). Twenty-five out of 64 cases (39%) were started on treatment between May and October 2020 and 39 of 64 cases (60%) between November 2020 and April 2021. The incidence of new cases of CPP through June 2022 continued to rise 6 months post the January 2022 peak in Covid-19 cases (data not shown).

## Discussion

CPP cases requiring GnRHa treatment significantly increased during the first year of Covid-19 compared to pre-Covid-19 years. Cases continued to rise after the first Covid year, and this has not been previously reported by others. No trend of increasing cases was observed for the 5 years prior to the pandemic. There was no delay in presentation or treatment initiation during Covid-19 based on age at diagnosis or degree of BA advancement. Time from diagnosis to onset of treatment and BMI were similar in both groups. In addition, a trend of increased CPP cases approximately 5–6 months following Covid-19 peaks was observed. Continued follow-up is needed before any conclusions can be made regarding a potential association.

The present results are consistent with those from other groups who demonstrated an increase in incidence of CPP during Covid-19, with a range from 1.6-fold to more than double (2, 4, 5, 7). Chioma et al. (4) and Umano (7) in Italy, and Acar (5) in Turkey also reported no difference in BMI, age at onset of puberty, or anthropometric parameters between their groups, as similarly shown by Ariza Jimenez in Spain (6). Others (14) evaluated the incidence of consults for suspected precocious or early puberty during Covid-19 pandemic in comparison to a pre-pandemic period and showed that the incidence during the pandemic was significantly higher, and there was no difference in anthropometric data between the time periods. Lifestyle questionnaires were used in a study (4), and it was concluded that those diagnosed with CPP during Covid-19 had a more sedentary lifestyle and more prolonged use of electronic devices in contrast to those without the diagnosis.

In this study, there was no difference in age at presentation for each of the groups. This is in contrast to the finding reported by Stagi et al. (2) that during the pandemic period, children presented at a younger age, a finding also presented by Orman et al. (15). Consistent across all studies published to date, including ours, is that degree of BA advancement was not different between children diagnosed with CPP before versus during the pandemic (2, 5, 7, 15). This is somewhat surprising since some of those studies did report more advanced Tanner stage at diagnosis (2, 3, 15).

The pandemic lockdown raised concerns regarding access to clinical care. At Rady Children's Hospital, telemedicine visits were initiated within a month of the start of the lock-down in San Diego and the endocrinology clinic re-opened for in-person visits shortly thereafter. The similar age at diagnosis and similar degree of bone age advancement during the Covid-19 year suggests that patients included in this study did not have a delay in access to care, nor a delay in diagnosis. In addition, there was no significant delay in onset of treatment. Interestingly, Orman et al. (15) showed shorter time to treatment during than before the pandemic and reported that during the pandemic, those diagnosed with CPP had a less advanced Tanner Stage than those diagnosed pre-pandemic.

Strengths of the current study include the historical perspective regarding number of children evaluated over 5 years prior to Covid-19, as well as a careful evaluation regarding the timing between diagnosis and GnRHa order, and timing between GnRHa order and onset of treatment before versus during the pandemic. Data collection was started for the pandemic year in May 2020 to eliminate the transition period from pre-Covid-19 to the pandemic. The same months were included for the pre-Covid group to control for any potential seasonal variation. Only cases of CPP who initiated treatment with GnRHa are reported. This is listed primarily as a strength, since this ensures inclusion of only those truly requiring treatment based on all parameters. Another strength of the present study is that it compared the incidence of newly diagnosed patients with CPP requiring treatment to the incidence of Covid-19 cases in the city. The first Covid peak noted in children occurred in July-August 2020 (13), and the peak of new CPP cases in this study was observed in November 2020, several months later. Another peak of Covid-19 cases occurred in January 2022 (13) and CPP cases have been rising since then, again, several months later.

A limitation of this study is the inability to determine how many children had a confirmed Covid-19 infection given the retrospective nature of the study as well as the fact that many children did not come to this institution for testing and others could have had asymptomatic infections. It has been previously reported, however, that very few individuals with recent diagnosis of CPP had confirmed Covid-19 infection and most had a negative history in terms of symptoms and diagnostic tests (16). Additionally, the study was retrospective at a single institution and extraction of newly diagnosed CPP patients was potentially limited by documentation in the EMR. Of note, some patients diagnosed during the Covid-19 year had onset of puberty prior to that year. This study reports incidence of patients newly diagnosed with CPP during the pandemic, not those with onset of puberty during that time.

Published studies evaluating the incidence of CPP during Covid-19 are from Italy, Turkey, Spain, and China. The present study is the first one published from a center in North America, and like others, shows a higher incidence of CPP during Covid-19. This highlights that CPP incidence increased regardless of the different quarantine and lock-down measures in different countries.

Factors influencing a higher incidence of CPP during the pandemic are unclear, and likely multifactorial. Hypotheses to date include direct relationship to the viral infection, the immune response, lifestyle changes, and emotional stress. Street et al. (16) published a comprehensive analysis of the potential causes for increased CPP during Covid-19, including direct effects of the virus on the central nervous system potentially altering gamma aminobutyric acid and N-methyl-D-aspartate, emotional and psychological factors involving higher production of catecholamines and dopamine, nutrition and physical activity changes affecting BMI or adiposity, changes in sleep habits associated with reduced melatonin, changes in the use of digital devices with subsequent reduction in reduced exposure to sunlight, and vitamin D deficiency and exposure to endocrine disruptors previously linked to precocious puberty. Interestingly, others (17) have reported menstrual cycle alterations in relation to Covid-19 pandemic and infection. In addition, there are many anecdotal reports regarding menstrual irregularities in relation to vaccination for Covid-19, all of which support possible hypothalamic-pituitary-gonadal axis and other hormonal pathway alterations in association with the Covid-19 pandemic. None of the children in the first year of the pandemic were vaccinated, so increased incidence of CPP is not related to the vaccine.

In conclusion, the number of cases of CPP initiating treatment with GnRHa was more than double during the first year of Covid-19 pandemic compared to a similar time period 1 year earlier as well as the previous 3 years. Age and degree of bone age advancement were not different between the 2 timeframes, suggesting there was no delay in presentation for medical care during Covid-19 for the patients studied. Additionally, there was no difference in the timing of treatment initiation. In this study, BMI was similar for both groups, so the higher incidence of CPP during the pandemic is unlikely to be related to nutrition or weight gain. Further study with larger cohorts is needed to understand the pathogenic factors contributing to a higher incidence of CPP in association with Covid-19 and whether this trend continues.

## Data Availability

The raw data supporting the conclusions of this article will be made available by the authors, without undue reservation.
